# Age, an Important Sociodemographic Determinant of Factors Influencing Consumers' Food Choices and Purchasing Habits: An English University Setting

**DOI:** 10.3389/fnut.2022.858593

**Published:** 2022-05-11

**Authors:** Daniel A. Ogundijo, Ayten A. Tas, Bukola A. Onarinde

**Affiliations:** ^1^National Centre for Food Manufacturing, University of Lincoln, Holbeach, United Kingdom; ^2^Department of Health Professions, Manchester Metropolitan University, Manchester, United Kingdom

**Keywords:** purchasing behaviors, sociodemographic characteristics, food choice, academic environment, nutrition labels

## Abstract

The purchasing behaviors of university staff (*n* = 188) and their use of nutrition labels in making food choices were investigated by an online survey. The age of the participants significantly impacted their purchasing behaviors. This effect was not observed with other sociodemographic characteristics studied (level of education, gender, employment status and ethnicity). The impact of age on the extrinsic factors affecting food choice (personal preference, previous knowledge, convenience, religion/beliefs) and intrinsic factors (quantity, country of origin, method of preparation/serving, fat, salt, protein and added sugar contents) were further explored. The use of nutrition labels among different age groups when buying for the first time was significant for breakfast cereals and fruit juices.

## Introduction

The choices of foods are becoming more complex, and consumers are faced with making informed decisions based on increasingly diverse factors. Several factors influencing consumers' food choices have been identified, such as the perceived healthiness of the food products, the health status of the consumers, religious beliefs (e.g., halal foods), philosophical beliefs, e.g., veganism and healthfulness, family/peer pressure, sustainability concerns, advertisements, quality of the food, ingredient list, cost/price, and availability (1–4).

Sociodemographic characteristics impact consumers' attitudes and behaviors toward foods, but these are often overlooked (4–6). Age, employment status, ethnicity, gender, and level of education are commonly used to analyse consumers' attitudes/habits and their understanding of nutritional knowledge in behavioral studies (7–9). Studies in non-academic settings established that males consumed more fast foods, paid less attention to nutrition, had lower nutrition knowledge, and used nutrition labels much less frequently than females (10–13). People with a high level of education usually had better nutrition knowledge, but the influence of knowledge on purchasing behaviors was rarely reported (14–16). Several studies investigated the effects of sociodemographic characteristics in academic settings (17, 18). These studies found that males were less knowledgeable about nutrition/healthy eating when compared to females, younger consumers (young adults) did not prepare foods from scratch as those in the middle ages and older, soft drink consumption was higher in males, and females had breakfast more often than males (17, 18).

One of the nutritional tools used in making informed purchasing decisions is nutrition labels, usually presented in various formats (18, 19). For example, different front of pack (FOP) labeling formats are used in countries across the world, such as the use of the UK government Traffic Light (TL) color labeling scheme, Australasia's Health Star Rating (HRS) system, Sweden's Keyhole labeling logo, France's Nutri-Score and the Chilean warning label (WL) (20, 21). Some FOP formats include interpretive information (such as Nutri-Score) that enables consumers to assess the product's healthiness. Others (such as Reference Intakes or Guideline Daily Amounts) provide factual information without any recommendation for the product's healthiness. Some formats (such as the TL) give a mix of factual and interpretive information (22).

Nutrition labels assist consumers in making informed food choices via the nutritional information they provide (20, 22). However, consumers' use of nutrition labels depends on the factors such as their level of understanding, nutrition knowledge and the extent to which they trust the information (23). The use of nutrition labels has been reported to vary across different settings and locations (24). For example, higher use of colored FOP labels was reported in countries such as Australia, New Zealand, the UK, and the USA; consumers in Canada and China preferred monochrome labels and those providing nutrient-specific information to the colored versions (25). While females used nutrition labels more than males in households, people with greater knowledge of healthy diets used more FOP labeling formats, particularly the TL scheme in academic settings (8, 26).

Purchasing/eating behaviors and consumers' use of nutrition labels have been investigated in different settings, such as academic, home, online environments, and health professionals (18, 24, 25, 27). Those studies looked at the use of nutrition labels by the consumers and how various demographic variables (such as age, gender, level of education, employment status etc.) affected it. For example, postgraduate certificate holders had higher knowledge and understanding of nutrition labels and used them more often (23, 28). However, to the best of our knowledge, studies investigating age as a factor affecting the use of nutrition labels in the UK academic settings are limited.

This study provided an in-depth discussion of the factors influencing consumers' food choices and purchasing habits in an academic environment. It specifically reviewed the effect of age in relation to extrinsic and intrinsic factors influencing purchasing decisions. It also investigated the frequency of nutrition label use by different age groups when buying for the first time.

## Methodology

A validated and structured online questionnaire (provided in Appendix 1) was used to collect information about food purchasing behaviors. The data was gathered between October 2020 and May 2021. A link to the survey was generated on the Qualtrics software and published on the university's “Staff News” website. One of the requirements to participate in the study was to identify as a university staff (either academic or administrative staff). Any potential participants that chose the options “student” or “I'm not a member of the University staff” were automatically screened out. Because the study aimed to investigate the food behaviors of university staff, students were excluded.

The survey investigated factors affecting consumers' food choices and buying habits and their use of nutrition information on food labels (Appendix 1). Face and content validity were used to test the reliability of the questions. Face validity was carried out to measure if the questions actually measured the study's objectives, and the content validity assessed whether every question was representative of all the aspects of the construct. To further test the validity and reliability, a pilot study, which was described elsewhere (9), was carried out. The questionnaire gathered data on sociodemographic variables (age, employment status, ethnic group, gender, and level of education) and food choice factors. A Likert scale was used to measure the participants' knowledge, perceptions, and behaviors around food choices. Three-point Likert scales were used to measure the knowledge and perceptions of participants on both the intrinsic and extrinsic food choice factors, and four-point scales were used to measure food behaviors. For example, the participants were assessed on how likely different extrinsic factors would influence their food purchasing decisions by choosing from the options “likely,” “neither likely nor unlikely,” or “unlikely.” Extrinsic (person-related) food factors assessed include convenience, availability, advertisement, previous knowledge about the food, consumer's health status, religion/beliefs, family/peer influence, tradition/culture, and personal preference. Intrinsic (food-related) factors included physical appearance (e.g., shape, color, size, taste, texture), price, brand, quality, quantity, healthiness, packaging, use by date, best before date, front of pack labeling, ingredient list and back of pack labeling.

Out of 2,250 staff that had access to the survey link, 242 used it to participate in the study; however, the responses of 188 participants (74.9%) were useable. The expected sample size for a reference population of 2,250 cases was 329 with a confidence level of 95% and a margin of error of 5%, assuming a dichotomous scale variable analyzed with p = q = 0.5 corresponding to a simple random sampling design. Initial power analysis with 5,000 simulations of the ordinal regressions (including simulated null effects for gender) had suggested that 329 participants would give 80% power in detecting a relationship with Spearman correlation coefficient, ρ = −0.15. With only 188 useable responses collected, a follow-up simulation showed that 80% power for Spearman correlations of ~ −0.2 or stronger could still be expected. This implied that the number of usable responses (*n* = 188) would allow estimating correlations higher than 0.2 belonging to a theoretical target population. The questions in the survey were regressed on age, with gender as a control variable, using proportional odds logistic regressions, with one regression for each survey response. Ordinal regression was carried out using R software version R 3.6.2 and package “MASS.” Statistical significance was declared for *p-*values below 0.05 (*p* < 0.05).

## Results

### Sociodemographic Characteristics of the Participants

As shown in Table 1, most of the participants were from the White ethnic background (89.89%) and people from Black, Asian, and other ethnic minorities comprised 9.04%. Using the Generations-Birth-Years^1^ age classification, most participants (42.55%) fell between 39 and 54, and only one participant preferred not to be identified with the stated age groups. The females constituted 63.83%, males 35.11%, and other participants who did not choose to identify with either male or female gender were 1.06%. More than half of the participants (54.26%) were postgraduate certificate holders, and 73.41% had at least a degree qualification. While 87.77% were in full-time employment, only 9.04% worked part-time.

**Table 1:** Sociodemographic characteristics of the participants (*n* = 188).

**Variable**	**Characteristics**	**Frequency**	**Percent (%)**
Age^*^	18–22	13	6.91
	23–38	64	34.04
	39–54	80	42.55
	55–73	30	15.96
	Prefer not to say	1	0.53
Gender	Male	66	35.11
	Female	120	63.83
	In another way	1	0.53
	Prefer not to say	1	0.53
Ethnicity	Asian or Asian British	6	3.19
	Black, African, Black British or Caribbean	6	3.19
	Mixed or multiple ethnic groups	4	2.13
	White (this includes any White background)	169	89.89
	Another ethnic group, for example, Arab	1	0.53
	Prefer not to say	2	1.06
Highest level of education	Secondary school	8	4.26
	College or vocational training	35	18.62
	Undergraduate	36	19.15
	Postgraduate	102	54.26
	Other	5	2.66
	Prefer not to say	2	1.06
Employment status	Employed / full time	165	87.77
	Employed / part-time	17	9.04
	Prefer not to say	6	3.19

* *Found to affect food choices and purchasing habits significantly, p < 0.05*.

### Extrinsic Factors Affecting Food Choice

**Figure 1:**
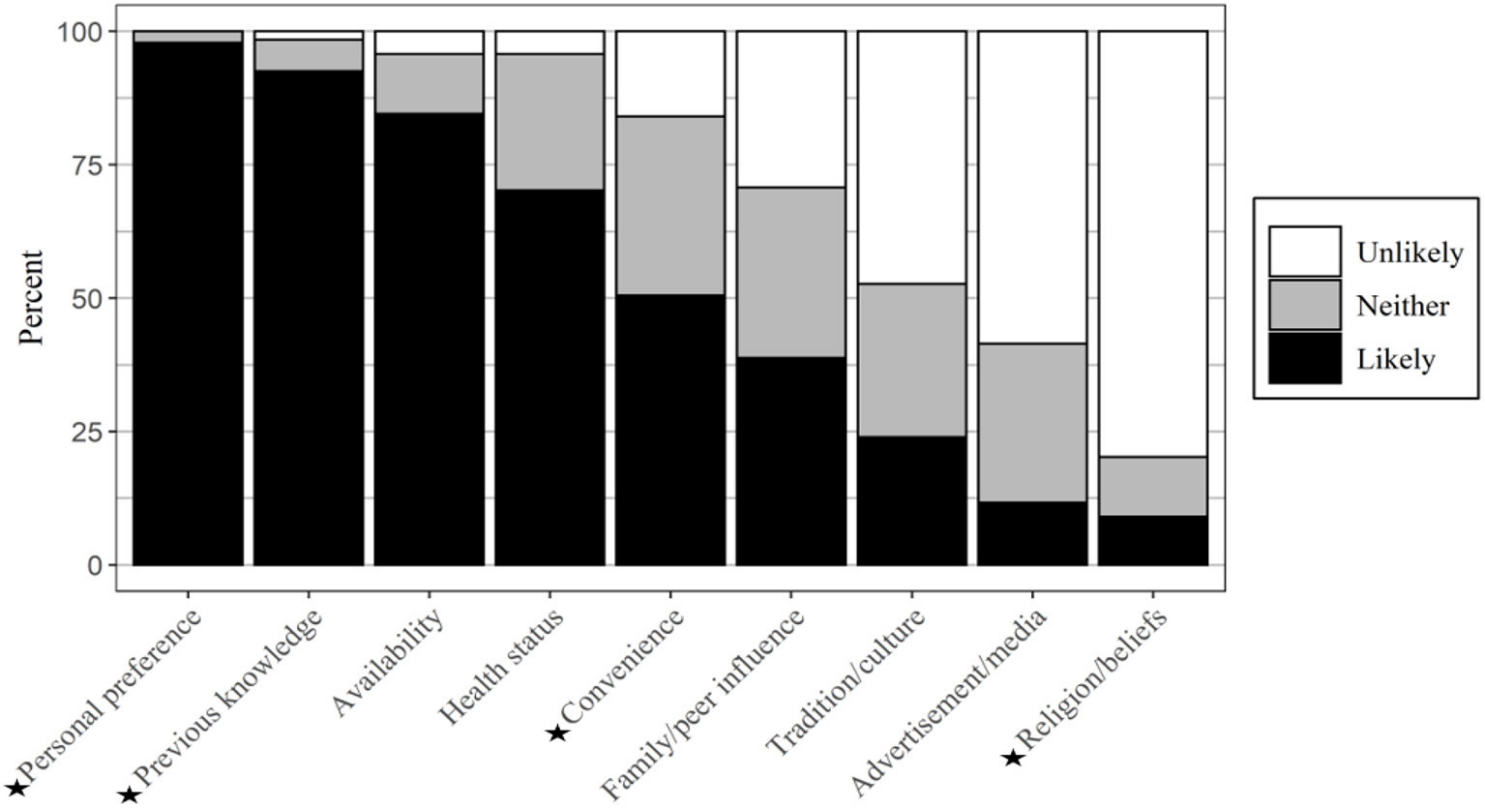
The extrinsic factors influencing food purchasing behaviors (*n* = 188). ⋆ Factors that showed a significant relationship with age.

**Figure 2:**
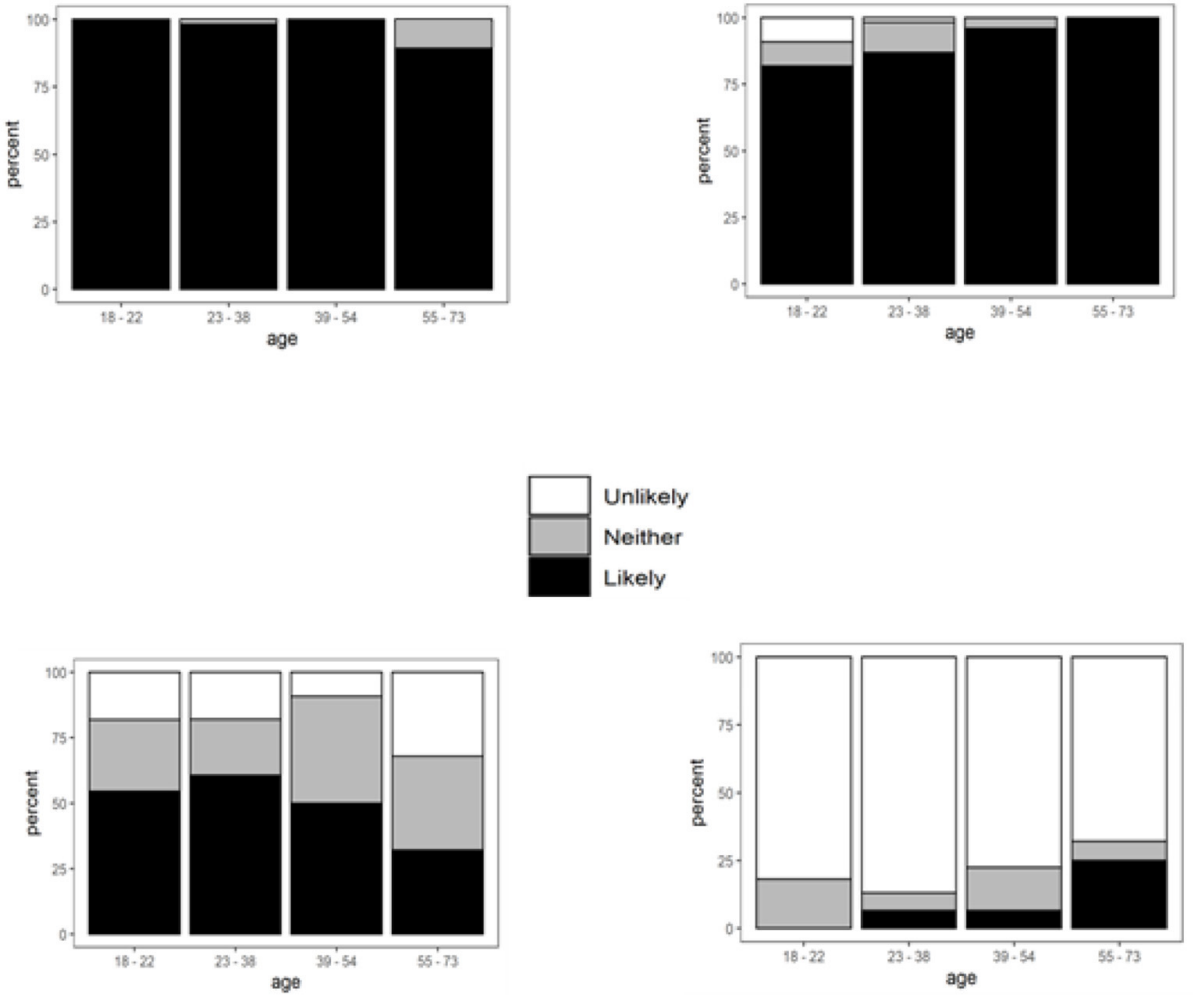
Extrinsic factors significantly affecting food purchasing behaviors when responses are grouped by age. Ordinal regression coefficients (log odds ratios per age category) ± *SE*: **(i)** Personal preference, *B* = −1.61 ± 0.82, *p* = 0.026. **(ii)** Previous knowledge about the food, *B* = 1.09 ± 0.40, *p* = 0.004. **(iii)** Convenience, *B* = −0.38 ± 0.19, *p* = 0.037. **(iv)** Religion/beliefs, *B* = 0.50 ± 0.24, *p* = 0.036.

Participants were asked to state how likely their food choices are influenced by nine extrinsic (personal, non-product related) factors (15, 26) (Figure 1). As presented in Table 2, a significant relationship with age (*p* < 0.05) was found with some of those factors: personal preference, previous knowledge about food, convenience, and religion/beliefs (Figure 2).

**Table 2:** Ordinal regression coefficients with significant *p-*values (*p* < 0.05).

Question^*^	**Parameter**	**Age.p**	**Gender.p**	**Age.b**	**Gender.b**	**Age.SE**	**Gender.SE**
A2.1	Breakfast cereals	0.002	0.381	0.865	−0.390	0.288	0.443
A2.13	Fruit juices	0.043	0.648	0.625	0.648	0.320	0.543
B2.1	Convenience	0.037	0.146	−0.384	−0.437	0.186	0.300
B2.4	Previous knowledge	0.003	0.749	1.091	0.202	0.398	0.637
B2.6	Religion/beliefs	0.036	0.396	0.498	−0.346	0.243	0.414
B2.9	Personal preference	0.026	0.076	−1.611	−1.927	0.823	1.188
B3.5	Quantity	0.001	0.439	−0.692	−0.234	0.191	0.302
B4.6	Country of origin	0.022	0.065	0.400	−0.545	0.176	0.296
B4.7	Preparation/serving instruction	0.015	0.452	−0.435	−0.231	0.180	0.307
B4.10	Added sugar	0.006	0.046	0.510	−0.611	0.187	0.307
B4.12	Salt	0.001	0.911	0.585	0.034	0.186	0.300
B4.14	Proteins	0.019	0.502	−0.411	−0.200	0.177	0.298
B4.16	Fat	0.049	0.365	0.354	−0.273	0.181	0.301

* *Items associated with age significantly (Appendix 1)*.

*Significant factors had the omnibus p-value below the 0.05 cut-off value of Age.p, Age.b represents the log odds ratios per age category; these were significant at p < 0.05. p: p-value, b, the log odd ratio per age category; SE, standard error*.

#### Personal Preference

Almost all participants (98%) said that how they made decisions when purchasing foods depended on their personal preferences, except a few participants aged 23–38 (1%) and 10% at the higher end of the age spectrum (55–73 years) [Figure 2(i)].

#### Previous Knowledge About the Food

Most participants across the age groups from 18 to 73 (93%) said that their previous knowledge of foods was likely to be a factor in choosing a particular food during shopping. Everyone in the 55+ age category (and the majority in 39–54) was likely to use their previous knowledge, apart from a few aged 18–38 [Figure 2(ii)].

#### Convenience

The 23–38 age category stated that convenience would influence their food shopping more than twice as often (61%) than did the 55–73 category (30%) [Figure 2(iii)].

#### Religion/Beliefs

Many participants (80%) claimed that religion or beliefs were unlikely to influence their purchasing decision. The number of participants who responded that this was likely increased with age. None of the participants aged 18–22 thought that religion/beliefs were likely to affect their decision on food choices compared with 27% of those aged 55–73 who stated the opposite [Figure 2(iv)].

### Intrinsic Factors Affecting Food Choice

**Figure 3:**
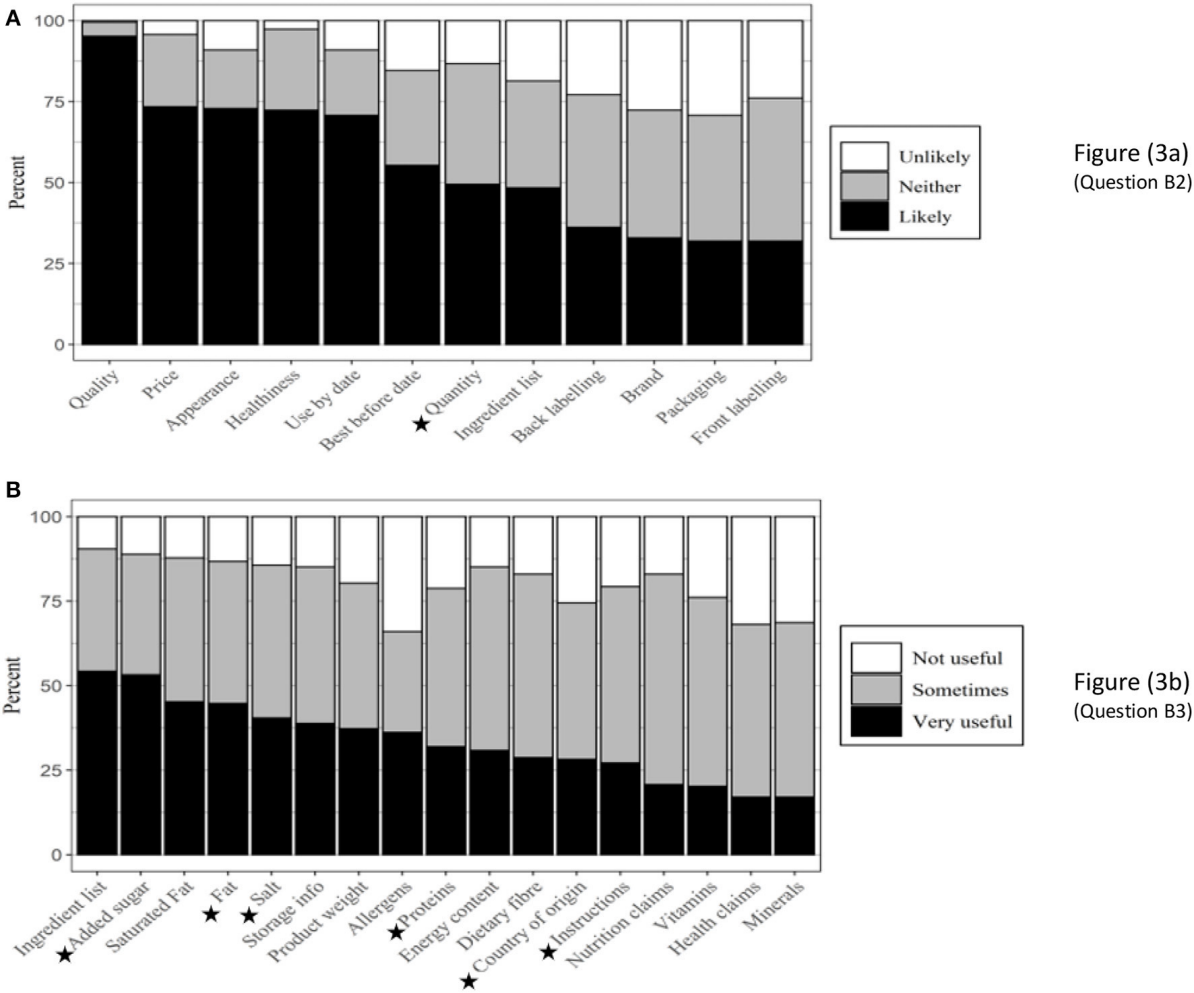
Intrinsic factors influencing food purchasing behaviors (*n* = 188). ⋆ Factors that showed a significant relationship with age. **(A)** (Question B2), **(B)** (Question B3).

Twenty-nine intrinsic factors (specific food attributes listed in questions B2 and B3) were presented to the participants (see Figures 3A,B for responses to questions B2 and B3, respectively). As presented in Table 2, seven showed a significant relationship with age: quantity, country of origin (COO), preparation/ serving instructions, added sugar, salt, protein, and fat contents (Figure 3).

#### Quantity

**Figure 4:**
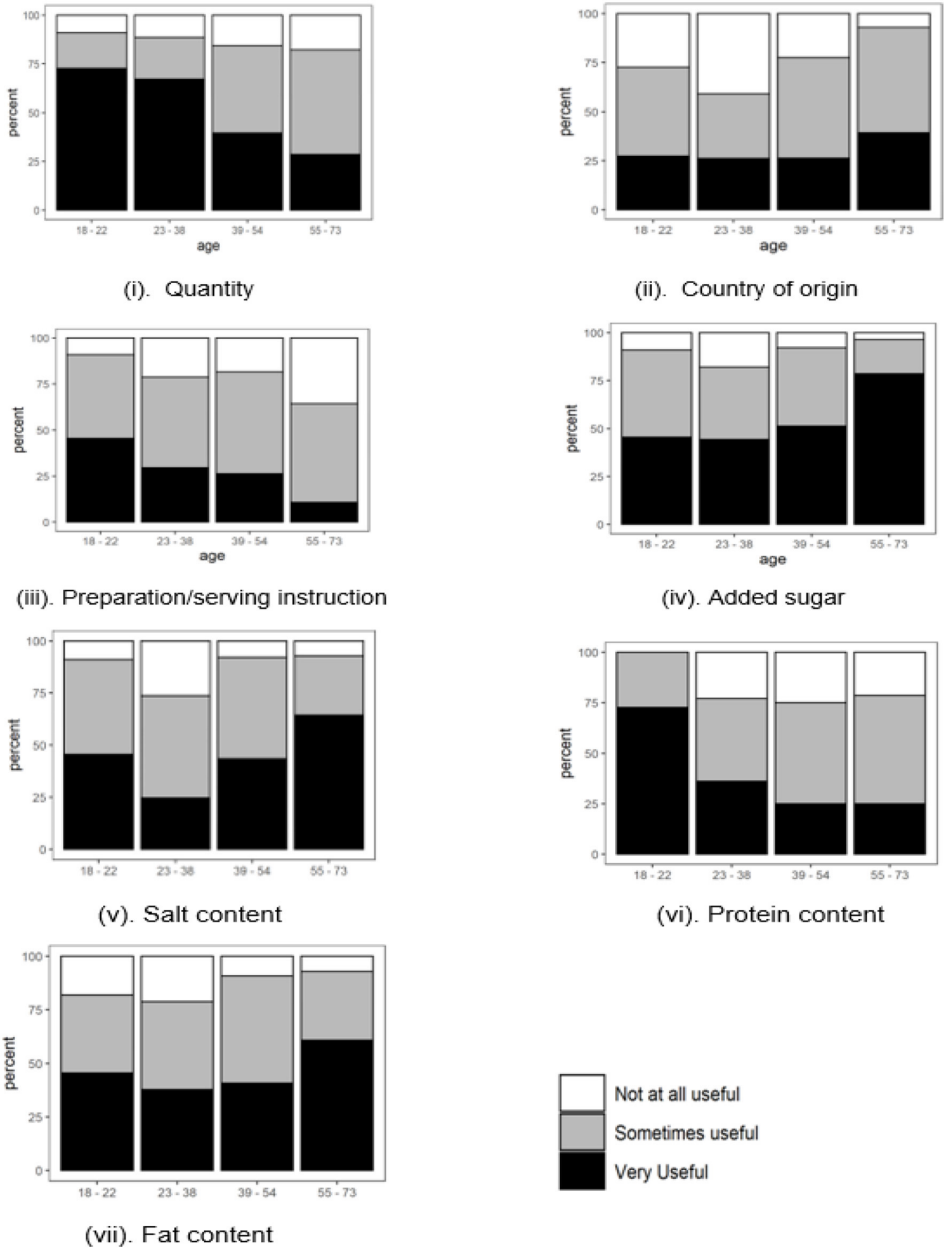
Intrinsic factors significantly affecting food purchasing behaviors when responses are grouped by age. Ordinal regression coefficients (log odds ratio per age category) ± *SE*: **(i)** Quantity, *B* = −0.69 ± 0.19, *p* < 0.001. **(ii)** Country of Origin, *B* = 0.40 ± 0.18, *p* = 0.022. **(iii)** Preparation/serving instructions, *B* = −0.44 ± 0.18, *p* = 0.015. **(iv)** Added sugar, *B* = 0.51 ± 0.19, *p* = 0.006. **(v)** Salt, *B* = 0.59 ± 0.19, *p* = 0.001. **(vi)** Protein, *B* = −0.41 ± 0.18, *p* = 0.019. **(vii)** Fat, *B* = 0.35 ± 0.18, *p* = 0.049.

The older the participants, the less likely the quantity of food products determined purchasing decisions [Figure 4(i)]. While 65% of the participants aged 18–38 stated that quantity was likely to influence their choices when shopping for foods, only around half as many (33%) of the 55–73 age group concurred.

#### Country of Origin

The information on COO was somewhat more important to the older age group, with 37% of 55–73-year-olds who found COO “very useful” compared with 26% of those who were younger [Figure 4(ii)].

#### Preparation/Serving Instructions

As presented in Figure 4(iii), the information on how a food product is prepared or served was more critical to the younger participants. The likelihood that preparation or serving instructions on food products would influence the purchasing decisions decreased steeply as consumers' age increased (46% in the youngest age group compared to 10% in the oldest).

#### Added Sugar

Added sugar content was considered far more useful by the oldest age group (80%) compared with the younger groups (48%); Figure 4(iv).

#### Salt Content

Salt showed a similar pattern to added sugar. Most participants in the 55–73 age group (76%) stated that the information on the amount of salt in the food products was “very useful” [Figure 4(v)]. The 23–38 age group had the lowest number of participants (22%) who stated that the amount of salt in the food products was “very useful” when making purchasing decisions. The highest number of participants who claimed that the salt content was “not at all useful” (24%) were also in the 23–38 age group.

#### Protein Content

The protein content of food products became less important to the participants in making purchasing decisions as they got older [Figure 4(vi)]. While many participants in the 18–22 age group (62%) found the information on protein content “very useful,” this figure was only 29% when the responses of other age groups were combined.

#### Fat Content

Figure 4(vii) shows that fat content showed a similar pattern with added sugar and salt. Most participants aged 55–73 found this information “very useful” (60%, compared to 41% from other age groups).

### Attitudes Toward the Use of Nutrition Labels

**Figure 5:**
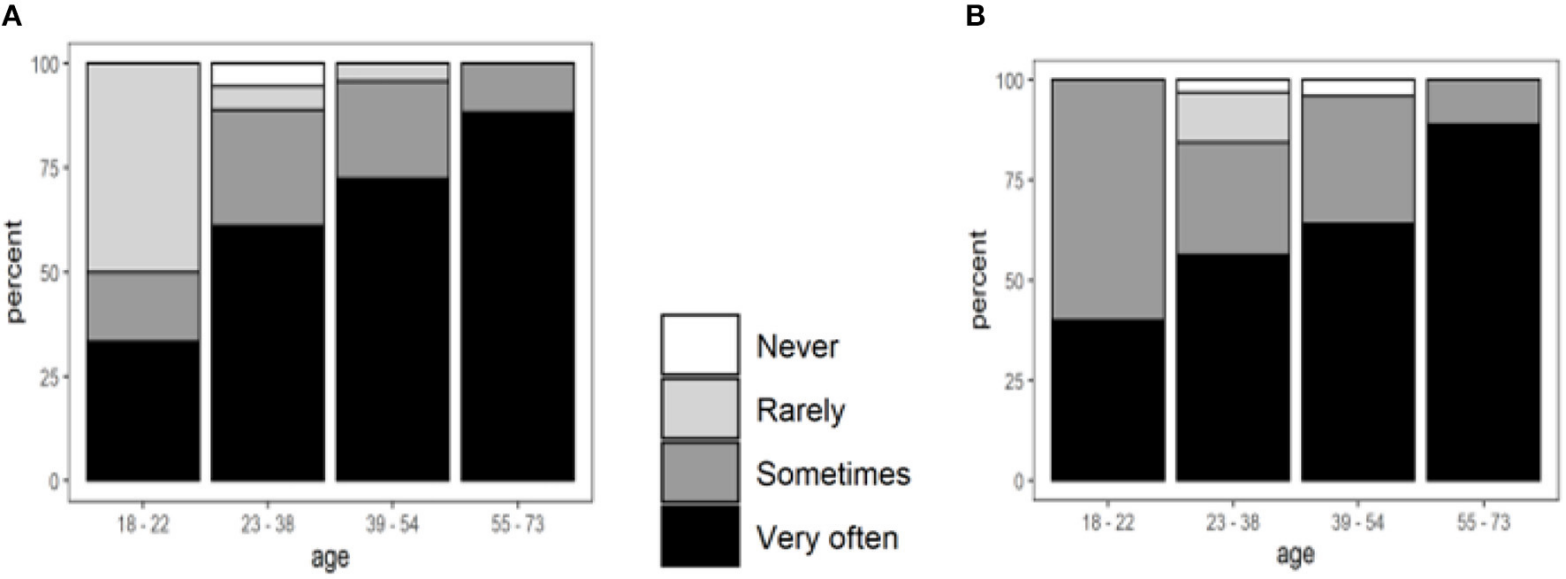
The frequency of the use of nutrition labels for breakfast cereals and fruit juices, grouped by age. Ordinal regression coefficients (log odds ratios per age category) ± *SE*: **(A)** Breakfast cereals, *B* = 0.86 ± 0.29, *p* = 0.002. **(B)** Fruit juices, *B* = 0.62 ± 0.32, *p* = 0.043.

Participants were asked about their use of nutrition labels for food categories such as breakfast cereals, dairy products, bread, biscuits/bakery products, etc., when purchasing food products for the first time (questions A1 and A2 in Appendix 1). The ages of the participants only affected the use of labels for breakfast cereals and juices significantly (Figure 5). Approximately 82% of the participants aged 55–73 checked the nutrition labels of breakfast cereals and fruit juices very often, but this decreased gradually with decreasing age.

## Discussion

### Extrinsic Factors

Personal preference was the most critical factor determining the purchasing behaviors across all age groups. Almost all participants below the age of 55 expressed that personal preference was likely to influence their food choices during shopping. This agrees with previous consumer studies, which reported that most consumers would purchase food products based on their experience of taste, texture, price, and satisfaction (27, 29, 30).

The previous knowledge about food products also had a significant influence on the purchasing decisions of the older participants. All participants in the most senior age group (55–73) -regardless of other demographic characteristics- would only purchase the foods they were familiar with. This finding where age greatly influenced the previous experience or familiarity with a food product did not agree with some of the existing studies. For example, rather than the age of the consumers, gender and education levels were reported to have a more significant impact on the previous experience with foods (18, 27, 31).

A limited number of studies undertaken in academic settings identified convenience, the healthiness of the food products, price, quality, and income levels as factors affecting food choices (23, 28, 32). The current study proposed that convenience was a significant factor affecting purchasing behavior in an academic setting, particularly for consumers aged 18–38.

This study could not confirm the possible impact of education level on purchasing decisions. Similarly, tradition/culture, the health status of the participants, advertisement/media, family/peer pressure, and the availability of food products did not significantly affect purchasing decisions.

### Intrinsic Factors

Many studies postulated that consumers might choose food products based on their quantity, but most did not claim quantity significantly affected food choice (18, 33–35). Although quantity can be an important determinant of choice among younger participants (as found in the current study), this may not apply to all foods. Allman-Farinelli et al. (36) recorded that young people preferred and overconsumed unhealthy foods because they were tastier than healthier alternatives. It is generally accepted that young adults (aged 18–38) favor some food products more than older adults, especially alcoholic and sweetened-sugar beverages (37–39).

The COO has also been reported as one of the factors for food choices in the UK. While some consumers did not consider the COO of some food products such as butter and coffee during shopping (40, 41), it was found that many UK consumers would buy foods based on this information (42–44). Existing studies established that ethnocentric consumers were biased about purchasing foreign food products, and some would not buy certain foods (such as infant foods) from countries such as China and Africa (45–47). Several reasons such as better taste, supporting local farmers, better quality, food safety, and environmental concerns were provided by consumers in the studies that looked at the influence of COO on food choices (48–50). The number of participants who claimed that the COO of food products was useful in making food choices in the current study (74.4%) was almost twice as what was reported nearly a decade ago by Kemp et al. (43). This increase may be due to the implications of the Covid-19 pandemic, Brexit, and concerns regarding the impact of the environment on the UK's food supply chain. Armstrong et al. (51) reported a growing distrust among UK consumers of the foods imported from the USA and China. Therefore, an increased number of consumers whose food choices are influenced by the COO are envisaged.

Evidence shows that UK consumers make informed food choices based on the cooking/preparation and serving instructions given on the food labels by the manufacturers (20, 22, 25). The current study proposed that the preparation/serving instructions on food labels were less useful to older adults. This could be because of the positive eating and cooking experience associated with the products over the years (31).

The participants' attitudes toward using the nutritional composition information (i.e., added sugar, fat and salt content) were similar to the findings reported by Bus and Worsley (52), where the information about these nutrients was beneficial for the elderly. This could be due to their increased awareness of healthy eating as they get older (26, 53, 54).

### Use of Labels

Contrary to our expectation that the level of education would influence the use of nutrition labels, age was the only significant demographic variable that seemed to affect it. The increased tendency to use nutrition labels with increasing age could be ascribed to the efforts to meet the specific dietary requirements as the metabolism declines with age (35).

The current study was consistent with a New Zealand study, which reported that the nutrition labels of foods with varying nutrient contents (such as convenience foods, breakfast cereals, snacks and bakery products) were more often checked during shopping than foods with homogenous nutrient content such as milk, fruits and vegetables (23). While Grunert et al. (26) reported that UK consumers, regardless of their profession and workplace, were more likely to use nutrition information on breakfast cereals during food shopping; Sah et al. (55) noted the use of fruit juice labels by the UK shoppers to ascertain the sugar contents. These studies had similar outcomes to the current one on using nutrition labels for breakfast cereals and fruit juices. Still, they were not undertaken in academic settings, and participants‘ age was not identified as a determining factor for food choice.

### Study Limitations and Implications to Research and Practice

One of the limitations of this study was the difficulty in data collection, which led to a low response rate. The use of incentives could have increased the participation and response rate. A higher power than the reported 80% in this study would have provided a more valid result; nonetheless, the total number of useable responses (*n* = 188) was sufficient to represent the study population. Many participants were from a White ethnic group with a possibility of having similar socioeconomic backgrounds; we could therefore say that diversity among the participants was limited, and the staff from ethnic minority groups were under-represented. The survey did not enquire about the participants' previous nutrition knowledge level, which might have impacted the results. The studies that critically investigate the associations between sociodemographic characteristics and factors influencing food choices in the UK are limited. Therefore, the results of this study would contribute to the existing knowledge base.

## Conclusion

While several sociodemographic variables determine food behaviors, this study suggested that age–but not level of education- was an important characteristic that predominantly impacted the factors influencing food purchasing decisions in an academic community. A more detailed study of specific factors affecting personal preferences when buying foods may help understand the complex decision-making processes and promote healthy eating habits. It was promising that most participants regarded nutritional composition information as useful. Using nutrition labels for food categories other than breakfast cereals and fruits juices would be highly desirable.

We suggest an effective use of nutrition labels on all food products, especially ready meals and snacks with high energy, salt, sugar, and saturated fat content that increase the risk of diet-related chronic diseases. We recommend a consistent assessment of the food purchasing habits/eating behaviors of people working in educational settings, which is often overlooked. Significant associations between sociodemographic variables and eating behaviors in such settings, which could be explored by complementing them with flexible methodologies such as classification and regression trees (CART), could help shape nutrition-related policies for academic communities.

## Data Availability Statement

The raw data supporting the conclusions of this article will be made available by the authors, without undue reservation.

## Author Contributions

DO, AT, and BO: conceptualization, investigation, methodology, validation, and visualization. DO: formal analysis, project administration, and roles/writing—original draft. AT and BO: supervision. DO and AT: writing—review and editing. All authors contributed to the article and approved the submitted version.

## Conflict of Interest

The authors declare that the research was conducted in the absence of any commercial or financial relationships that could be construed as a potential conflict of interest.

## Publisher's Note

All claims expressed in this article are solely those of the authors and do not necessarily represent those of their affiliated organizations, or those of the publisher, the editors and the reviewers. Any product that may be evaluated in this article, or claim that may be made by its manufacturer, is not guaranteed or endorsed by the publisher.

## Footnotes

^1^Generation Z, Generation X, Millennials, Baby Boomers https://jasondorsey.com/about-generations/generations-birth-years/.

## References

[1] Puddephatt JA Keenan GS Fielden A Reaves DL Halford JCG Hardman CA. 'Eating to survive': a qualitative analysis of factors influencing food choice and eating behaviour in a food-insecure population. Appetite. (2020) 147:104547. 10.1016/j.appet.2019.104547 31812558

[2] Kershaw KN Klikuszowian E Schrader L Siddique J Van Horn L Womack VY. Assessment of the influence of food attributes on meal choice selection by socioeconomic status and race/ethnicity among women living in Chicago, USA: a discrete choice experiment. Appetite. (2019) 139:19–25. 10.1016/j.appet.2019.04.003 30974181 PMC7656662

[3] Scaglioni S De Cosmi V Ciappolino V Parazzini F Brambilla P Agostoni C. Factors influencing children's eating behaviours. Nutrients. (2018) 10:706. 10.3390/nu10060706 29857549 PMC6024598

[4] Kaya IH. Motivation factors of consumers' food choice. Food Nutr Sci. (2016) 7:149–54. 10.4236/fns.2016.73016

[5] Piernas C Popkin BM. Increased portion sizes from energy-dense foods affect total energy intake at eating occasions in US children and adolescents: patterns and trends by age group and sociodemographic characteristics, 1977-2006. Am J Clin Nutr. (2011) 94:1324–32. 10.3945/ajcn.110.008466 21918222 PMC3192477

[6] Acton RB Vanderlee L Roberto CA Hammond D. Consumer perceptions of specific design characteristics for front-of-package nutrition labels. Health Educ Res. (2018) 33:167–74. 10.1093/her/cyy006 29514225

[7] Wardle J Haase AM Steptoe A Nillapun M Jonwutiwes K Bellisle F. Gender differences in food choice: the contribution of health beliefs and dieting. Ann Behav Med. (2004) 27:107–16. 10.1207/s15324796abm2702_5 15053018

[8] Stran KA Knol LL. Determinants of food label use differ by sex. J Acad Nutr Diet. (2013) 113:673–9. 10.1016/j.jand.2012.12.014 23402696

[9] Ogundijo DA Tas AA Onarinde BA. Exploring the impact of COVID-19 pandemic on eating and purchasing behaviours of people living in England. Nutrients. (2021) 13:1499. 10.3390/nu13051499 33946799 PMC8146722

[10] Stobbelaar DJ Casimir G Borghuis J Marks I Meijer L Zebeda S. Adolescents' attitudes towards organic food: a survey of 15- to 16-year old school children. Int J Consum Stud. (2007) 31:349–56. 10.1111/j.1470-6431.2006.00560.x

[11] Grunert KG Hieke S Wills J. Sustainability labels on food products: consumer motivation, understanding and use. Food Policy. (2014) 44:177–89. 10.1016/j.foodpol.2013.12.001

[12] Cooke R Papadaki A. Nutrition label use mediates the positive relationship between nutrition knowledge and attitudes towards healthy eating with dietary quality among university students in the UK. Appetite. (2014) 83:297–303. 10.1016/j.appet.2014.08.039 25218881

[13] Basfirinci C Cilingir UkZ. Does country of origin matter for chocolate? ethnocentrism, involvement, and perceived risk for Turkish university students. J Food Prod Mark. (2020) 26:144–84. 10.1080/10454446.2020.1740128

[14] Parmenter K Waller J Wardle J. Demographic variation in nutrition knowledge in England. Health Educ Res. (2000) 15:163. 10.1093/her/15.2.163 10751375 PMC4344545

[15] Contento IR. Nutrition education: linking research, theory, and practice. Asia Pac J Clin Nutr. (2008) 17(Suppl. 1):176–9. 18296331

[16] Alsaffar AA. Exploring the level of nutrition knowledge and influencing factors in a Turkish community sample. Int peer-reviewed. J Nutr Res. (2014) 1:45. 10.17362/DBHAD.201428960

[17] Mantzari E Pechey R Codling S Sexton O Hollands GJ Marteau TM. The impact of 'on-pack' pictorial health warning labels and calorie information labels on drink choice: a laboratory experiment. Appetite. (2020) 145:104484. 10.1016/j.appet.2019.104484 31626833 PMC8161725

[18] Lima JPM Costa SA Brandão TRS Rocha A. Food consumption determinants and barriers for healthy eating at the workplace—a university setting. Foods. (2021) 10:695. 10.3390/foods10040695 33805929 PMC8064356

[19] Tas AA Ahmed H Alnatour G Koca K. “Healthy Snack” intervention to Improve the nutritional knowledge of university students. Adv Nutr Food Sci. (2020) 2020:1–8. Available online at: https://kosmospublishers.com/healthy-snack-intervention-to-improve-the-nutritional-knowledge-of-university-students/

[20] Pérusse-Lachance É Tremblay A Drapeau V. Lifestyle factors and other health measures in a Canadian university community. Appl Physiol Nutr Metab. (2010) 35:498–506. 10.1139/H10-035 20725116

[21] Cecchini M Warin L. Impact of food labelling systems on food choices and eating behaviours: a systematic review and meta-analysis of randomised studies. Obes Rev. (2016) 17:201–10. 10.1111/obr.12364 26693944

[22] Rramani Q Krajbich I Enax L Brustkern L Weber B. Salient nutrition labels shift peoples' attention to healthy foods and exert more influence on their choices. Nutr Res. (2020) 80:106–16. 10.1016/j.nutres.2020.06.013 32739728

[23] Ogundijo DA Tas AA Onarinde BA. An assessment of nutrition information on front of pack labels and healthiness of foods in the United Kingdom retail market. BMC Public Health. (2021) 21:1–10. 10.1186/s12889-021-10255-4 33550987 PMC7868120

[24] Feunekes GIJ Gortemaker IA Willems AA Lion R van den Kommer M. Front-of-pack nutrition labelling: testing effectiveness of different nutrition labelling formats front-of-pack in four European countries. Appetite. (2008) 50:57–70. 10.1016/j.appet.2007.05.009 17629351

[25] European Heart Network (EHN). Front-of-Pack (FOP) Nutrition Labelling – European Heart Network Position. Brussels: European Heart Network AISBL (2020).

[26] Grunert KG Fernández-Celemín L Wills JM Storcksdieck Genannt Bonsmann S Nureeva L. Use and understanding of nutrition information on food labels in six European countries. J Public Heal. (2009) 18:261–77. 10.1007/s10389-009-0307-0 21124644 PMC2967247

[27] Lundeberg PJ Graham DJ Mohr GS. Comparison of two front-of-package nutrition labeling schemes, and their explanation, on consumers' perception of product healthfulness and food choice. Appetite. (2018) 125:548–56. 10.1016/j.appet.2018.02.027 29496604

[28] Van der Horst K Bucher T Duncanson K Murawski B Labbe D. Consumer understanding, perception and interpretation of serving size information on food labels: a scoping review. Nutrients. (2019) 11:2189. 10.3390/nu11092189 31514395 PMC6770558

[29] Ni Mhurchu C Eyles H Jiang Y Blakely T. Do nutrition labels influence healthier food choices? analysis of label viewing behaviour and subsequent food purchases in a labelling intervention trial. Appetite. (2018) 121:360–5. 10.1016/j.appet.2017.11.105 29191745

[30] Pettigrew S Dana LM Talati Z Tian M Praveen D. The role of colour and summary indicators in influencing front-of-pack food label effectiveness across seven countries. Public Health Nutr. (2021) 24:3566–70. 10.1017/S1368980020004966 33317658 PMC10195333

[31] Bassola B Tommasi V Bonetti L Bauer S Lusignani M. Nurses' knowledge about malnutrition in older people: a multicenter cross-sectional study. Nutrition. (2020) 1:78. 10.1016/j.nut.2020.110947 32861178

[32] Moore SG Donnelly J Jones S Cade J. Use and understanding of current UK nutrition label information. Proc Nutr Soc. (2018) 77:176. 10.1017/S0029665118001829

[33] Lindseth G. Factors affecting graduating nurses' nutritional knowledge: implications for continuing education. J Contin Educ Nurs. (1997) 28:245–51. 10.3928/0022-0124-19971101-04 9416044

[34] Abood DA Black DR Feral D. Nutrition education worksite intervention for university staff: application of the health belief model. J Nutr Educ Behav. (2003) 35:260–7. 10.1016/S1499-4046(06)60057-2 14521826

[35] Jeruszka-Bielak M Kollajtis-Dolowy A Santoro A Ostan R Berendsen AAM Jennings A. Are nutrition-related knowledge and attitudes reflected in lifestyle and health among elderly people? a study across five European countries. Front Physiol. (2018) 9:994. 10.3389/fphys.2018.00994 30108512 PMC6079245

[36] Allman-Farinelli M Partridge SR Roy R. Weight-related dietary behaviors in young adults. Curr Obes Rep. (2016) 5:23–9. 10.1007/s13679-016-0189-8 26811006

[37] Chong FS Farmer LJ Hagan TDJ Speers JS Sanderson DW Devlin DJ. Regional, socioeconomic and behavioural- impacts on consumer acceptability of beef in Northern Ireland, Republic of Ireland and Great Britain. Meat Sci. (2019) 154:86–95. 10.1016/j.meatsci.2019.04.009 31022586

[38] Shafie FA Rennie D. Consumer perceptions towards organic food. Procedia Soc Behav Sci. (2012) 49:360–7. 10.1016/j.sbspro.2012.07.034

[39] Prescott J Young O O'Neill L Yau NJN Stevens R. Motives for food choice: a comparison of consumers from Japan, Taiwan, Malaysia and New Zealand. Food Qual Prefer. (2002) 13:489–95. 10.1016/S0950-3293(02)00010-1

[40] Ahmed ZU Johnson JP Yang X Fatt CK Teng HS Boon LC. Does country of origin matter for low-involvement products? Int Mark Rev. (2004) 21:102–20. 10.1108/02651330410522925

[41] Trinh G Corsi A Lockshin L. How country of origins of food products compete and grow. J Retail Consum Serv. (2019) 49:231–41. 10.1016/j.jretconser.2019.03.027

[42] Barnett J Vasileiou K Gowland MH Raats MM Lucas JS. Beyond labelling: what strategies do nut allergic individuals employ to make food choices? a qualitative study. PLoS ONE. (2013) 8:e55293. 10.1371/journal.pone.0055293 23383141 PMC3558473

[43] Kemp K Insch A Holdsworth DK Knight JG. Food miles: do UK consumers actually care? Food Policy. (2010) 35:504–13. 10.1016/j.foodpol.2010.05.011

[44] Felzensztein C Dinnie K. The Effects of Country of Origin on UK Consumers' Perceptions of Imported Wines. Taylor & Francis Group (2008). Available online at: https://www.tandfonline.com/doi/abs/10.1300/J038v11n04_08 (accessed November 22, 2021).

[45] Yang Q Zhang Z Gregg EW Flanders WD Merritt R Hu FB. Added sugar intake and cardiovascular diseases mortality among us adults. JAMA Intern Med. (2014) 174:516–24. 10.1001/jamainternmed.2013.13563 24493081 PMC10910551

[46] Weatherell C Tregear A Allinson J. In search of the concerned consumer: UK public perceptions of food, farming and buying local. J Rural Stud. (2003) 19:233–44. 10.1016/S0743-0167(02)00083-9

[47] Herbert G Butler L Kennedy O Lobb A. Young UK adults and the 5 A DAY campaign: perceived benefits and barriers of eating more fruits and vegetables. Int J Consum Stud. (2010) 34:657–64. 10.1111/j.1470-6431.2010.00872.x

[48] Buckley M Cowan C McCarthy M O'Sullivan C. The convenience consumer and food-related lifestyles in great Britain. J Food Prod Mark. (2005) 11:3–25. 10.1300/J038v11n03_0226993575

[49] Freedman M Rubinstein R. Obesity and food choices among faculty and staff at a large urban university. J Am Coll Health. (2010) 59:205–10. 10.1080/07448481.2010.502203 21186451

[50] Mantzari E Vasiljevic M Turney I Pilling M MT. Impact of warning labels on sugar-sweetened beverages on parental selection: an online experimental study. Prev Med Reports. (2018) 12:259–67. 10.1016/j.pmedr.2018.10.016 30406003 PMC6215029

[51] Armstrong B Reynolds C Wilson AM Xhakollari V. China and the USA, a higher perceived risk for UK consumers in a post COVID-19 food system: the impact of country of origin and ethical information on consumer perceptions of food. Emerald Open Res. (2020) 2:35. 10.35241/emeraldopenres.13711.1

[52] Bus AEM Worsley A. Consumers' health perceptions of three types of milk: a survey in Australia. Appetite. (2003) 40:93–100. 10.1016/S0195-6663(03)00004-7 12781158

[53] Han E Powell LM. Consumption patterns of sugar-sweetened beverages in the United States. J Acad Nutr Diet. (2013) 113:43–53. 10.1016/j.jand.2012.09.016 23260723 PMC3662243

[54] Chryssochoidis G Krystallis A Perreas P. Ethnocentric beliefs and country-of-origin (COO) effect: Impact of country, product and product attributes on Greek consumers' evaluation of food products. Eur J Mark. (2007) 41:1518–44. 10.1108/03090560710821288

[55] Sah A Hillenbrand C Vogt J. Visible sugar : salient sugar information impacts health perception of fruit juices but only when motivated to be responsible and not when motivated to enjoy. Appetite. (2021) 164:105262. 10.1016/j.appet.2021.105262 33862190

