# Supplementary material for: Age, an Important Sociodemographic Determinant of Factors Influencing Consumers' Food Choices and Purchasing Habits: An English University Setting
Source: Front Nutr. 2022 May 11;9:858593. doi: 10.3389/fnut.2022.858593 (PMC9132226; doi:10.3389/fnut.2022.858593)
Supplement: Supplementary file 1 [file Presentation_1.pdf]

Appendix 1: Study questionnaire

Exploring consumer knowledge and the use of nutritional labels in making healthier food choices.

We would like to invite you to take part in our research study. Before you proceed, we would like you to understand why the research is being carried out.

What is the purpose of the study? This study explores the perception by food manufacturers and retailers on the use of nutrition labels and how they have responded or are responding to the increased public interest in making informed food choices.

Do I have to take part? No, it is up to you to decide whether to take part. If you do decide to take part, you will be asked to sign a consent form. You are free to withdraw at any time, without giving any reason, and without your legal rights being affected.

What will happen to me if I take part? You will be asked to fill in a self-reporting questionnaire regarding your experiences and perceptions on the nutrition labelling culture in your organisation. Your participation will take approximately 12 to 15 minutes. There are no right or wrong answers: we are simply interested in your honest opinions. Your participation is completely anonymous.

Will I receive any incentive after I take part? Participants will not be paid or offered any other incentive to participate in the study.

What are the possible disadvantages and risks of taking part? The project is considered as low risk based on the topics and methods.

Will my taking part in the study be kept confidential? Your information will be kept completely confidential, and we shall follow all appropriate ethical and legal practices as required by the University of Lincoln

Privacy notice: The University's Research Participant Privacy notice explains how we will be using information from you in order to undertake this study and will be the data controller for this study. This means that we are responsible for looking after your information and using it properly

What will happen to the results of the research study? The results of the research study will only be used for statistical analysis and academic purposes.

Who is organising and funding the research? This research is organised by the University of Lincoln and self-funded by the researcher.

Who has reviewed the study? All research conducted by the University of Lincoln is looked at by an independent group of people, called the Research Ethics committee to protect your interests. The ethics committee approval reference for this study is 2151.

What if there is a problem? If you have a concern about any aspect of this study, you should ask to speak to the researcher who will do his best to answer your questions. The researcher contact detail is provided at the end of this information sheet. If you remain unhappy and wish to complain formally, you can do this by contacting ethics@lincoln.ac.uk.

If you feel that we have let you down in relation to your information protection rights, then please contact the Information Compliance team by email on compliance@lincoln.ac.uk or by post at Information Compliance, Secretariat, University of Lincoln, Brayford Pool, Lincoln, LN6 7TS.

You can also make complaints directly to the Information Commissioner's Office (ICO). The ICO is the independent authority upholding information rights for the UK. The website is ico.org.uk and their telephone helpline number is 0303 123 1113.

Should you have any queries or concerns, please contact Daniel Ogundijo.

Email: DOgundijo@lincoln.ac.uk

Are you over 18 years old?

○ Yes

○ No

Declaration of consent

I understand that my participation is voluntary and that I am free to withdraw at any time without giving any reason, without my legal rights being affected.

Please accept to give consent.

○ Accept Import Questions From...

Section A. Consumer's use of nutrition information and nutrition labels

A1. For which of the following food categories would you usually check the labels? Please select all that apply

- ☐ Breakfast cereals e.g., porridge, muesli. granola, cornflakes
- ☐ Dairy products e.g., milk, yoghurts, cream, cheese, kefir, whey, fromage-frais, butter
- ☐ Bread, biscuits/bakery products
- ☐ Crisps and snacks
- ☐ Ready meals e.g., sandwiches, pastas, soups
- ☐ Spreads such as jams, jellies, marmite and
- ☐ Vegetable oils
- ☐ Herbs and spices
- ☐ Fruit juices
- ☐ Non-alcoholic drinks Group A, e.g., coffee, tea, chocolate, cocoa products, etc.
- ☐ Non-alcoholic drinks Group B, e.g. carbonated drink, fizzy drink
- ☐ Alcoholic drinks e.g., beer, cider, ☐ spirits,

margarine
- ☐ Beans and other pulses e.g., lentils
- ☐ Canned or jarred food products such as meat, fish, fruits, vegetables
- ☐ Eggs
- ☐ Nuts and seeds

vodka, gin, tequila, rum, whisky, brandy, etc.
- ☐ Food supplements and products that boost immunity
- ☐ None of the above

A2. How often do you check the nutrition labels of foods in these categories especially when buying for the first time? Please select the most appropriate option in the drop-boxes

»Breakfast cereals e.g., porridge, muesli, granola, cornflakes

» Dairy products e.g., milk, yoghurt, cream, cheese, kefir, whey, fromage-frais, butter

» Bread, biscuits/bakery products

» Crisps and snacks

» Ready meals e.g., sandwiches, pastas, soups

» Spreads such as jams, jellies, marmite and margarine

» Beans and other pulses e.g., lentils

» Canned or jarred food products such as meat, fish, fruits, vegetables

» Eggs

» Nuts and seeds

» Vegetable oils

» Herbs and spices

» Fruit juices

» Non-alcoholic drinks Group A, e.g., coffee, tea, chocolate, cocoa products, etc.

» Non-alcoholic drinks Group B, e.g., carbonated drink, fizzy drink,

» Water e.g., sparkling water, mineral water

» Alcoholic drinks e.g., beer, cider, spirits, vodka, gin, tequila, rum, whisky, brandy, etc.

» Food supplements and products that boost immunity

» None of the above

A3. How easy to understand do you normally find the nutrition information on a food product label?

- Easy
- Slightly easy
- Slightly difficult
- Difficult

A4. How do you think nutrition information is helpful to decide the healthiness of food products?

- Very helpful
- Slightly helpful
- Not at all helpful
- I don't know

Section B. Factors affecting consumer's food choices and buying habits

This section covers how Covid-19 pandemic has affected your food shopping habit, and the possible drivers for making decisions on food choices

B1. The decision on buying foods in your household is usually made by

- Myself
- Partner
- Partner and myself
- Other family member
- Everyone in the house
- Other

B2. How likely will each of the following factors influence your decision-making during food shopping? Please select the most appropriate option in the drop-boxes

Convenience

Availability

Advertisement/media

Previous knowledge about the food

Health status

Religion/beliefs

Family/peer influence

Tradition/culture

Personal preference

B3. How likely will each of the following factors influence your decision-making during food shopping? Please select the most appropriate option in the drop-boxes

Physical appearance (e.g. shape, colour, size, taste, texture)

Price

Brand

Quality

Quantity

Healthiness

Packaging

Use by date

Best before date

Front of pack labelling

Ingredient list

Back of pack labelling

B4. How useful is the following information in making decisions at the point of purchase during your food shopping? Please select the most appropriate option in the drop-boxes

Nutrition claims

Health claims

Ingredient list

Allergen declaration

Product weight

Country of origin

Preparation/serving instruction

Storage information

Energy content

Added sugar

Saturated Fat

Salt

Vitamins

Proteins

Dietary fibre

Fat

Minerals

Section C. Demographic information

We would like to know about you. This data will be kept anonymously and only be used for statistical analysis.

C1. Please select the range of your age

- 18 - 22
- 23 - 38
- 39 - 54
- 55 - 73
- Prefer not to say

C2. How would you describe yourself?

○ Male

○ Female

○ In another way

○ Prefer not to say

If you describe your gender with another term, please provide this here:

C3. How would you describe your ethnic group?

○ Asian or Asian British. This includes any Asian background, for example, Bangladeshi, Chinese, Indian, Pakistani ○ Black, African, Black British or Caribbean. This includes any Black background

○ Mixed or multiple ethnic groups. This includes any Mixed background

○ White. This includes any White background

○ Another ethnic group. This includes any other ethnic group, for example, Arab

○ Prefer not to say

C4. Please select your highest level of education.

○ Secondary school

○ College or vocational training

○ Undergraduate

○ Postgraduate

○ Other

○ Prefer not to say

C5. What best describes your current employment status?

○ Employed / full time

○ Employed / part-time (less than 37 hours a week)

○ Prefer not to say
